# Enhanced inulinase production by *Streptomyces* sp. in solid state fermentation through statistical designs

**DOI:** 10.1007/s13205-012-0112-2

**Published:** 2013-01-03

**Authors:** M. Dilipkumar, M. Rajasimman, N. Rajamohan

**Affiliations:** 1 Department of Chemical Engineering, Annamalai University, Annamalainagar, Tamilnadu 608002 India; 2 Department of Chemical Engineering, Sohar University, Sohar, 311 Oman

**Keywords:** Inulinase, Copra waste, *Streptomyces* sp., Optimization, RSM

## Abstract

In this work, inulinase was produced by solid state fermentation by *Streptomyces* sp. using copra waste as carbon source. The nutrients were screened by Plackett–Burman design. From the pareto chart it was found that the nutrients, namely, soya bean cake, MgSO_4_·7H_2_O and (NH_4_)_2_SO_4_ were found to be most significant nutrient components. Hence, these three components were selected for further optimization using central composite design (CCD) in response surface methodology (RSM). The optimum conditions were soya bean cake: 0.05711 g/gds, MgSO_4_·7H_2_O: 0.00063 g/gds and (NH_4_)_2_SO_4_: 0.00772 g/gds. Under these optimized conditions, the production of inulinase was found to be 131 U/gds. The constants in the Michaelis–Menten equation were evaluated and high *R*^2^ value implies the fitness of the model.

## Introduction

Inulin (a polyfructan consisting of linear β-2,l linked fructose) is a linear biopolymer made up of fructose residues linked by β-2,1 bonds (Vandamme and Derycke 1983) that constitute the storage of carbohydrate in the roots and tubers of different plants (Cazetta et al. 2005; Singh et al. 2006). Its hydrolysis by microbial inulinases (β-2,1-d-fructan fructanohydrolase, EC 3.2.1.7) appears to be an interesting alternative for the production of high fructose, calorie reduced sweeteners, which are produced mainly by enzymatic isomerization of prehydrolyzed corn starch (Vandamme and Derycke 1983; Bajpai and Margaritis 1986; Manzoni and Cavazzoni 1992; Pandey et al. 1999; Rocha et al. 2006). Inulinase is highly prevalent in higher plants and microorganisms. This enzyme can be used for isolation of fructose from raw plants: Jerusalem artichoke (*Helianthus tuberosus*), dahlia (*dahlia*), elecampane (*Inula helenium*), chicory (*Cichorium*), dandelion (*Taraxacum*) and burdock (*Arctia*) (Kovaleva et al. 2009). Inulinase which catalyzes hydrolysis of inulin is produced by many microorganisms, such as K*luyveromyces, Aspergillus, Staphylococcus, Xanthomona, Pseudomonas, Candida kefyr, Debaryomyces cantarelli*, *Penicillium*, and *Streptomyces* sp. (Pandey et al. 1999). Solid state fermentation (SSF) offers numerous advantages for the production of bulk chemicals and enzymes due to low-cost substrates, simplified downstream and environmental-friendly process, reduced energy requirement, reduced wastewater produced, high yield of fermentation products, high volumetric productivity, increased product recovery, and simplicity of bioreactor design compared to submerged fermentation (SmF) (Pandey 2003). The use of the sequential strategy of the experimental design is a useful tool for process optimization. In the present work, the optimization of inulinase production by *Streptomyces* sp. using copra waste as substrate was carried out using a sequential strategy of the experimental design. Copra waste is a cheap substrate which is abundantly available in southern part of India (Kerala and Tamilnadu). Hence, it was selected as substrate for the production of inulinase.

## Materials and methods

### Actinomycete strain

Microorganism used in this work was well preserved in the laboratory. *Streptomyces* sp. MTCC-3119 was a stock of the Microbial Type Culture collection Centre (MTCC), Chandigarh, India. The strain was maintained on solid medium at 5 °C. The medium composition was composed of the following: yeast extract 4.0 g; malt extract 10.0 g; glucose 4.0 g; agar 20.0 g; distilled water 1.0 L; pH 7.2–7.4. Cells were harvested from slants and used to inoculate liquid medium.

### Solid state fermentation

Commercial quality copra waste (coconut oil cake) was procured from the local market and used as a substrate for inulinase production. Fermentation was carried out in Erlenmeyer flasks (250 ml) with 10 g of copra waste, supplemented with nutrient concentrations defined by the experimental design. Moisture was adjusted to 65 %. Each flask was covered with hydrophobic cotton and autoclaved at 121 °C for 20 min. After cooling, each flask was inoculated with 2 ml of the suspension previously prepared and incubated for 96 h in a chamber with temperature and humidity control. The whole contents were incubated at 37 °C (Sharma et al. 2006). During the preliminary screening process, the experiments were carried out for 5 days, and it was found that at the 24th h, the maximum production occurs. Hence, experiments are carried out for 24 h. All the experiments were carried out in triplicate and the average values were reported.

### Extraction of inulinase

After fermentation, 10 volumes of distilled water were added to the fermented matter, and the contents were agitated for 30 min at 200 rpm on a rotary shaker (at 28 °C). Then the sample was centrifuged at 15,000 rpm for 20 min, and the supernatants were analyzed by DNS method (Miller 1959).

### Optimization of inulinase production

Response surface methodology (RSM) consists of a group of empirical techniques used for evaluation of relationship between cluster of controlled experimental factors and measured response. A prior knowledge with understanding of the related bioprocesses is necessary for a realistic modeling approach. To determine which variables significantly affect inulinase production by *Streptomyces* sp., Plackett–Burman design (PBD) was used. Eighteen variables (Table 1) were screened in 20 experimental runs (Table 2), and insignificant ones were eliminated to obtain a smaller, manageable set of factors. The low level (−1) and high level (+1) of each factor are listed in (Table 1). The statistical software package “Design Expert 7.1.5” was used for analyzing the experimental data. Once the critical factors were identified through the screening, the central composite design (CCD) was used to obtain a quadratic model, consisting of factorial trials and star points to estimate quadratic effects and central points to estimate the pure process variability with inulinase production as response. RSM was employed to optimize the selected three significant nutrient components, namely, soya bean cake, MgSO_4_·7H_2_O and (NH_4_)_2_SO_4_ which enhances the inulinase production. The three independent variables were studied at five different levels (Table 3), and sets of 20 experiments were carried out (Table 4). The statistical software package “Design Expert 7.1.5” was used to analyze the experimental data. All variables were taken at a central coded value of zero. The minimum and maximum ranges of variables investigated were listed in (Table 3). Upon the completion of experiments, the average maximum inulinase activity was taken as the response (*Y*). A multiple regression analysis of the data was carried out for obtaining an empirical model that relates the response measured to the independent variables. A second-order polynomial equation is

**Table 1:** Nutrient screening using a Plackett–Burman design

Nutrient code	Nutrient	Levels (g/10 gds)	
		Low (−1)	High (+1)
A	Yeast extract	0.01	0.05
B	Beef extract	0.05	0.15
C	MnSO_4_·7H_2_O	0.10	0.50
D	K_2_HPO_4_	0.02	0.07
E	Soya bean cake	0.40	0.80
F	MgSO_4_·7H_2_O	0.002	0.012
G	NH_4_Cl	0.01	0.03
H	KCl	0.005	0.015
J	(NH_4_)_2_HPO_4_	0.05	0.30
K	NH_4_NO_3_	0.05	0.10
L	ZnSO_4_·7H_2_O	0.10	0.50
M	(NH_4_)_2_SO_4_	0.06	0.10
N	Corn steep liquor	0.40	0.80
O	Peptone	0.05	0.15
P	Dextrose	0.10	0.30
Q	FeSO_4_·7H_2_O	0.0005	0.002
R	KH_2_PO_4_	0.10	0.60
S	Urea	0.10	0.30

**Table 2:** Plackett–Burman experimental design matrix for screening of important variables for inulinase production using *Streptomyces* sp.

Run no.	A	B	C	D	E	F	G	H	J	K	L	M	N	O	P	Q	R	S	Inulinase (U/gds)
1	1	−1	1	−1	1	1	1	1	−1	−1	1	1	−1	1	1	−1	−1	−1	35.32
2	1	−1	1	1	1	1	−1	−1	1	1	−1	1	1	−1	−1	−1	−1	1	38.43
3	−1	−1	−1	−1	1	−1	1	−1	1	1	1	1	−1	−1	1	1	−1	1	21.32
4	−1	−1	−1	−1	−1	−1	−1	−1	−1	−1	−1	−1	−1	−1	−1	−1	−1	−1	53.00
5	−1	−1	−1	1	−1	1	−1	1	1	1	1	−1	−1	1	1	−1	1	1	61.45
6	1	1	−1	1	1	−1	−1	−1	−1	1	−1	1	−1	1	1	1	1	−1	45.30
7	−1	−1	1	1	−1	1	1	−1	−1	−1	−1	1	−1	1	−1	1	1	1	28.45
8	−1	1	−1	1	1	1	1	−1	−1	1	1	−1	1	1	−1	−1	−1	−1	40.65
9	1	−1	−1	−1	−1	1	−1	1	−1	1	1	1	1	−1	−1	1	1	−1	31.62
10	−1	1	−1	1	−1	1	1	1	1	−1	−1	1	1	−1	1	1	−1	−1	32.64
11	1	−1	1	1	−1	−1	−1	−1	1	−1	1	−1	1	1	1	1	−1	−1	81.72
12	−1	1	1	1	1	−1	−1	1	1	−1	1	1	−1	−1	−1	−1	1	−1	42.33
13	1	1	1	−1	−1	1	1	−1	1	1	−1	−1	−1	−1	1	−1	1	−1	52.31
14	1	1	−1	−1	1	1	−1	1	1	−1	−1	−1	−1	1	−1	1	−1	1	33.49
15	1	−1	−1	1	1	−1	1	1	−1	−1	−1	−1	1	−1	1	−1	1	1	62.51
16	1	1	1	1	−1	−1	1	1	−1	1	1	−1	−1	−1	−1	1	−1	1	74.84
17	−1	−1	1	−1	1	−1	1	1	1	1	−1	−1	1	1	−1	1	1	−1	33.43
18	1	1	−1	−1	−1	−1	1	−1	1	−1	1	1	1	1	−1	−1	1	1	47.23
19	−1	1	1	−1	−1	−1	−1	1	−1	1	−1	1	1	1	1	−1	−1	1	82.54
20	−1	1	1	−1	1	1	−1	−1	−1	−1	1	−1	1	−1	1	1	1	1	45.89

**Table 3:** Ranges of the independent variables used in RSM

Variables	Levels (g/gds)					
	Code	−1.68	−1	0	+1	+1.68
Soya bean cake	X_1_	0.04	0.05	0.06	0.07	0.08
MgSO_4_·7H_2_O	X_2_	0.0002	0.0004	0.0007	0.00095	0.0012
(NH_4_)_2_SO_4_	X_3_	0.006	0.007	0.008	0.009	0.01

**Table 4:** Central composite design (CCD) of factors in coded levels with enzyme activity as response

Run no.	*X*_1_	*X*_2_	*X*_3_	Inulinase activity (U/gds)	
				Experimental	Predicted
1	0.00	1.68	0.00	41.65	41.82
2	1.00	1.00	1.00	50.66	48.51
3	1.00	−1.00	−1.00	42.63	50.14
4	0.00	0.00	0.00	120.76	120.91
5	0.00	0.00	−1.68	102.35	90.18
6	0.00	0.00	0.00	124.65	120.91
7	0.00	0.00	0.00	123.34	120.91
8	0.00	0.00	0.00	121.66	120.91
9	0.00	0.00	0.00	126.34	120.91
10	−1.00	−1.00	−1.00	102.64	98.25
11	1.00	−1.00	1.00	59.74	41.99
12	0.00	0.00	0.00	122.34	120.91
13	0.00	−1.68	0.00	59.76	67.01
14	1.00	1.00	−1.00	69.23	68.66
15	−1.00	1.00	1.00	48.75	33.62
16	0.00	0.00	1.68	39.78	59.65
17	−1.00	1.00	−1.00	51.34	61.77
18	1.68	0.00	0.00	45.74	48.95
19	−1.68	0.00	0.00	72.86	76.87
20	−1.00	−1.00	1.00	88.34	82.10

1where *Y* is the measured response, β_0_ is the intercept term, β_*i*_ are linear coefficients, β_*ii*_ are quadratic coefficient, β_*ij*_ are interaction coefficient, and *X*_*i*_ and *X*_*j*_ are coded independent variables. The optimal concentrations of the critical variables were obtained by analyzing 3D plots. The statistical analysis of the model was represented in the form of analysis of variance (ANOVA).

### Assay of enzyme activity

Enzymes were assayed by measuring the concentration of reducing sugars released from sucrose. The reaction mixture containing 1 mL of diluted crude enzyme and 4 mL of 2 % sucrose (dissolved in 0.1 M acetate buffer, pH 5.0) was incubated at 50 °C. After incubating for 30 min, aliquots of 0.5 mL were withdrawn and increase in reducing sugar was estimated by a 3,5-dinitrosalicylic acid method (Miller 1959) using calibration curve obtained with a standard solution of fructose (Uzunova et al. 2002). Absorbance was read at 575 nm. A higher absorbance indicated a high level of reducing sugar produced and consequently a high enzyme activity. One unit of inulinase activity (U) was defined as the amount of enzyme, which forms 1 μmol fructose per min. Results of the determination of inulinase activity were presented in units of activity/gram of dry substrate (U/gds).

### Michaelis–Menten kinetics

In this study, the kinetics of the inulinase production was carried out using Michaelis–Menten equation. For carrying out kinetics, experiments were carried out at various substrate concentrations viz. 6, 8, 10, 12, and 14 g. The Michaelis–Menten equation is2

*V*_0_: Reaction rate, M/min
*V*_max_: Maximum reaction rate, M/min
*K*_m_: Inverse of enzyme affinity, mM
*S*: Substrate concentration, mM

A plot of time versus production was drawn. From the plot, the values of dP/dt was found for various points. Using the different dP/dt values for the various substrates concentrations, the constants *V*_max_ and *K*_m_ were evaluated using CF tool in MATLAB 7.0.

## Results and discussion

### Composition of copra waste

The composition of copra waste was analyzed and were given as: protein: 16.7 %; moisture: 7.6 %; fat: 3.2 %; ash: 4.2 %; crude fiber: 11.2 %; lipids: 11.8 %; crude cellulose: 17.8 %.

### Screening of nutrients by PBD

**Fig. 1:**
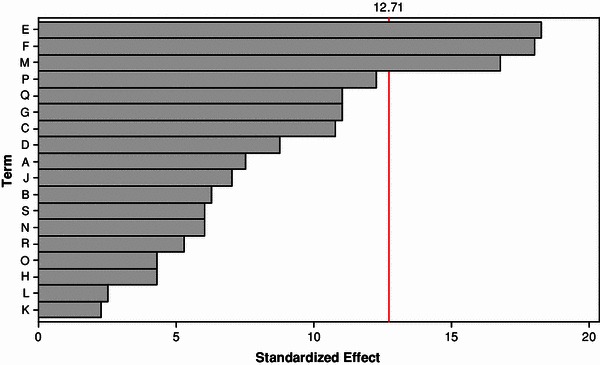
Pareto chart showing the effect of media components on inulinase activity using *Streptomyces* sp. (*A* yeast extract; *B* beef extract; *C* MnSO_4_·7H_2_O; *D* K_2_HPO_4_; *E*-soya bean cake; *F* MgSO_4_·7H_2_O; *G* NH_4_Cl; *H* KCl; *I* (NH_4_)_2_HPO_4_; *J* NH_4_NO_3_; *K* ZnSO_4_·7H_2_O; *L* (NH_4_)_2_SO_4_; *M* corn steep liquor; *N* peptone; *O* dextrose; *P* FeSO_4_·7H_2_O; *Q* KH_2_PO_4_; *R* urea)

Experiments were carried out based on Plackett–Burman design and the results obtained were given in Table 2. From the table, it was observed that there is a wide variation in inulinase activity. This variation reflected the importance of optimization to attain higher productivity. From the Pareto chart (Fig. 1), the nutrients, soya bean cake, MgSO_4_·7H_2_O and (NH_4_)_2_SO_4_ were found to be significant for the production of inulinase by S*treptomyces* sp. using copra waste. Hence, these nutrients were selected for further optimization using RSM to maximize the production of inulinase.

### Optimization of nutrients by CCD

CCD was used to determine the optimum conditions for the inulinase production using *Streptomyces* sp. The range and levels of factors [soya bean cake, MgSO_4_·7H_2_O and (NH_4_)_2_SO_4_] were given in Table 3. Twenty experiments were performed at different combinations. The predicted and observed responses along with design matrix were presented in Table 4. The results were analyzed by ANOVA. The second-order regression equation provides the inulinase activity as the function of soya bean cake, MgSO_4_·7H_2_O and (NH_4_)_2_SO_4_. This can be presented in terms of coded factors as:3where *Y* is the inulinase activity (U/gds), *X*_1_, *X*_2_ and *X*_3_ are soya bean cake, MgSO_4_·7H_2_O and (NH_4_)_2_SO_4_, respectively.

ANOVA for the response surface was shown in Table 5. The model *F* value of 14.29 implies that the model is significant. There is only a 0.01 % chance that a “Model *F* value” this large could occur due to noise. Values of “Prob > *F*” less than 0.05 indicate model terms were significant. Values greater than 0.1 indicate the model terms were not significant. In the present work, all the linear, interactive effects of *X*_1_*X*_2_ and square effects of *X*_1_, *X*_2_ and *X*_3_ were significant for inulinase production. The coefficient of determination (*R*^2^) for inulinase activity was calculated as 0.9279, which is very close to 1 and can explain up to 92.79 % variability of the response. The predicted *R*^2^ value of 0.7640 is in reasonable agreement with the adjusted *R*^2^ value of 0.8329. An adequate precision value greater than 4 is desirable. The adequate precision value of 10.043 indicates an adequate signal and suggests that the model can be used to navigate the design space.

**Table 5:** Analysis of variance (ANOVA) for response surface quadratic model for the production of inulinase

Source	Coefficient factor	Sum of squares	DF	*F*	*P* > *F*
Model	120.914	19,438.58	9	14.29	0.0001
*X*_1_	−8.30414	941.76	1	6.23	0.0316
*X*_2_	−7.48871	765.89	1	5.07	0.0481
*X*_3_	−9.07624	1,125.03	1	7.44	0.0212
*X*_1_ × *X*_2_	13.7500	1,512.50	1	10.01	0.0101
*X*_1_ × *X*_3_	2.00000	32.00	1	0.21	0.6552
*X*_2_ × *X*_3_	−3.00000	72.00	1	0.48	0.5057
*X*_1_ × *X*_1_	−20.5071	6,060.51	1	40.10	<0.0001
*X*_2_ × *X*_2_	−23.5123	7,966.94	1	52.72	<0.0001
*X*_3_ × *X*_3_	−16.2644	3,812.23	1	25.23	0.0005
Residual		1,511.17	10		
Lack of fit		1,404.34	5	13.15	0.0067
Pure error		106.83	5		
Cor total		20,949.75	19		

SD: 12.29; *R*^2^: 92.79 %; Mean: 79.75; Adj *R*^2^: 83.29 %; CV %: 15.41; Pred *R*^2^: 76.40 %; Adeq precision: 10.043

**Fig. 2:**
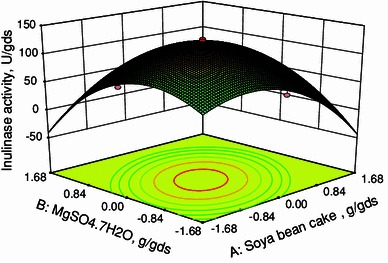
3D plot showing the effect of soya bean cake and MgSO_4_·7H_2_O on inulinase activity

**Fig. 3:**
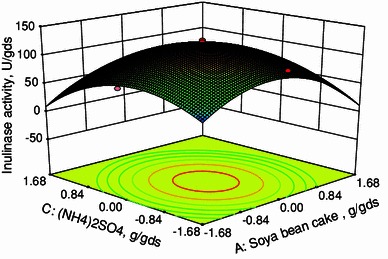
3D plot showing the effect of soya bean cake and (NH_4_)_2_SO_4_ on inulinase activity

**Fig. 4:**
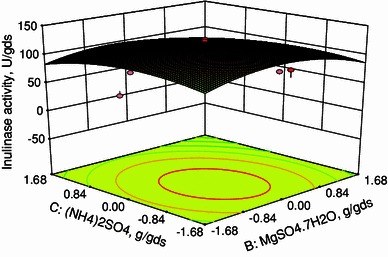
3D plot showing the effect of MgSO_4_·7H_2_O and (NH_4_)_2_SO_4_ on inulinase activity

Equation (2) can be used to predict the inulinase production within the limits of the experimental factors. The interactive effects of variables on inulinase production are studied by plotting 3D surface curves against any two independent variables, while keeping the other variables at its central (0) level. The 3D curves of the calculated response (inulinase production) and contour plots from the interactions between the variables were shown in Figs. 2, 3, and 4. Figure 2 shows the dependency of inulinase on soya bean cake and MgSO_4_·7H_2_O. The inulinase activity increases with increase in soya bean cake concentration up to 0.05711 g/gds and thereafter inulinase activity decreases with further increase in soya bean cake concentration. The same trend was observed in Fig. 3. Increase in MgSO_4_·7H_2_O resulted increase in inulinase activity up to 0.00063 g/gds. This is evident from Figs. 2 and 4. Figures 3 and 4 shows the dependency of inulinase activity on (NH_4_)_2_SO_4_. The effect of (NH_4_)_2_SO_4_ on inulinase observed was similar to soya bean cake. At low concentrations of nutrients the inulinase activity was found to be low due to lack of nutrients. At higher concentrations, these nutrients may affect the growth of microorganisms. Hence at higher concentrations, the inulinase activity decreases. The optimum conditions for the maximum production of inulinase were: soya bean cake: 0.05711 g/gds, MgSO_4_·7H_2_O: 0.00063 g/gds, and (NH_4_)_2_SO_4_: 0.00772 g/gds.

Validation of the experimental model was tested by carrying out the batch experiment under optimal operation conditions. Three repeated experiments were performed and the results were compared. The inulinase activity obtained from experiments was very close to the actual response predicted by the regression model, which proved the validity of the model. At these optimized conditions the maximum inulinase activity was found to be 131 U/gds. In Run no 6, a maximum production of inulinase was found to be 124.65 U/gds and it was increased to 131 U/gds at the optimized condition.

The results obtained in this work were compared with others and were given in Table 6. From the table it was inferred that the inulinase activity obtained in this study were comparable with others.

**Table 6:** Comparison of inulinase production

Substrate	Microorganisms	Inulinase activity (U/gds)	References
Wheat bran	*Staphylococcus* sp.	107.64	Selvakumar and Pandey 1999
	*Kluyveromyces marxianus*	122.88	
Dry sugarcane bagasse	*Kluyveromyces marxianus*	391.9	Mazutti et al. 2006
Sugarcane bagasse	*Kluyveromyces marxianus*	250	Mazutti et al. 2007
Wheat bran and rice husk	*Cryptococcus aureus*	436.2	Sheng et al. 2009
Sugar cane bagasse	*Kluyveromyces marxianus*	396.6	Bender et al. 2008
Wheat bran and rise bran	*Pichia guilliermondii,*	291.0	Guo et al. 2009
Pressmud	*Streptomyces* sp.	89	Dilipkumar et al. 2011
Copra waste	*Streptomyces* sp.	131	This work

### Kinetics study

In this study, Michaelis–Menten equation was used to describe the kinetics of the inulinase enzyme production. The constants *V*_max_ and *K*_m_ in Michaelis–Menten equation were found using MATLAB tool. The values were *V*_max_ = 25.43 M/min and *K*_m_ = 14.32 mM. A lower value of Michaelis–Menten constant indicates a strong affinity of the reaction. Normally *K*_m_ value for industrial enzymes lies in the range of 0.01–100 mM (Fullbrook 1996). In this study, the *K*_m_ value was found to be 14.3 mM. The results obtained were in accordance with Mazutti et al. 2007; Catana et al. 2005). Mazutti et al. (2007) obtained a *K*_m_ value of 7.10 mM for the inulinase production using *K.marxianus* and Catana et al. (2005) obtained a *K*_m_ value of 82 mM. The *R*^2^ value was 0.9245, which was greater than 0.9 shows the fitness of the model for inulinase production.

## Conclusions

Plackett–Burman design was used to test the relative importance of medium components on inulinase production. Among the variables, soya bean cake, MgSO_4_·7H_2_O and (NH_4_)_2_SO_4_ were found to be the most significant variables. From further optimization studies, the optimized values of the variables for inulinase production were as follows: soya bean cake: 0.05711 g/gds, MgSO_4_·7H_2_O: 0.00063 g/gds, and (NH_4_)_2_SO_4_: 0.00772 g/gds. This study showed that the copra waste constitutes a good carbon source for the production of inulinase. Using the optimized conditions, the produced activity reaches 131 U/gds. The results show a close concordance between the expected and obtained activity level. Michaelis–Menten kinetics was applied to the experimental data and a high *R*^2^ shows the aptness of the model for inulinase production.
